# Supplementary material for: Modification of the Neck Linker of KIF18A Alters Microtubule Subpopulation Preference
Source: bioRxiv. 2023 May 2:2023.05.02.539080. Preprint. [Version 1] doi: 10.1101/2023.05.02.539080 (PMC10187232; doi:10.1101/2023.05.02.539080)
Supplement: Supplement 8 [file NIHPP2023.05.02.539080v1-supplement-8.pdf]

bioRxiv preprint doi: https://doi.org/10.1101/2023.05.02.539080; this version posted May 2, 2023. The copyright holder for this preprint (which was not certified by peer review) is the author/funder, who has granted bioRxiv a license to display the preprint in perpetuity. It is made available under aCC-BY 4.0 International license.

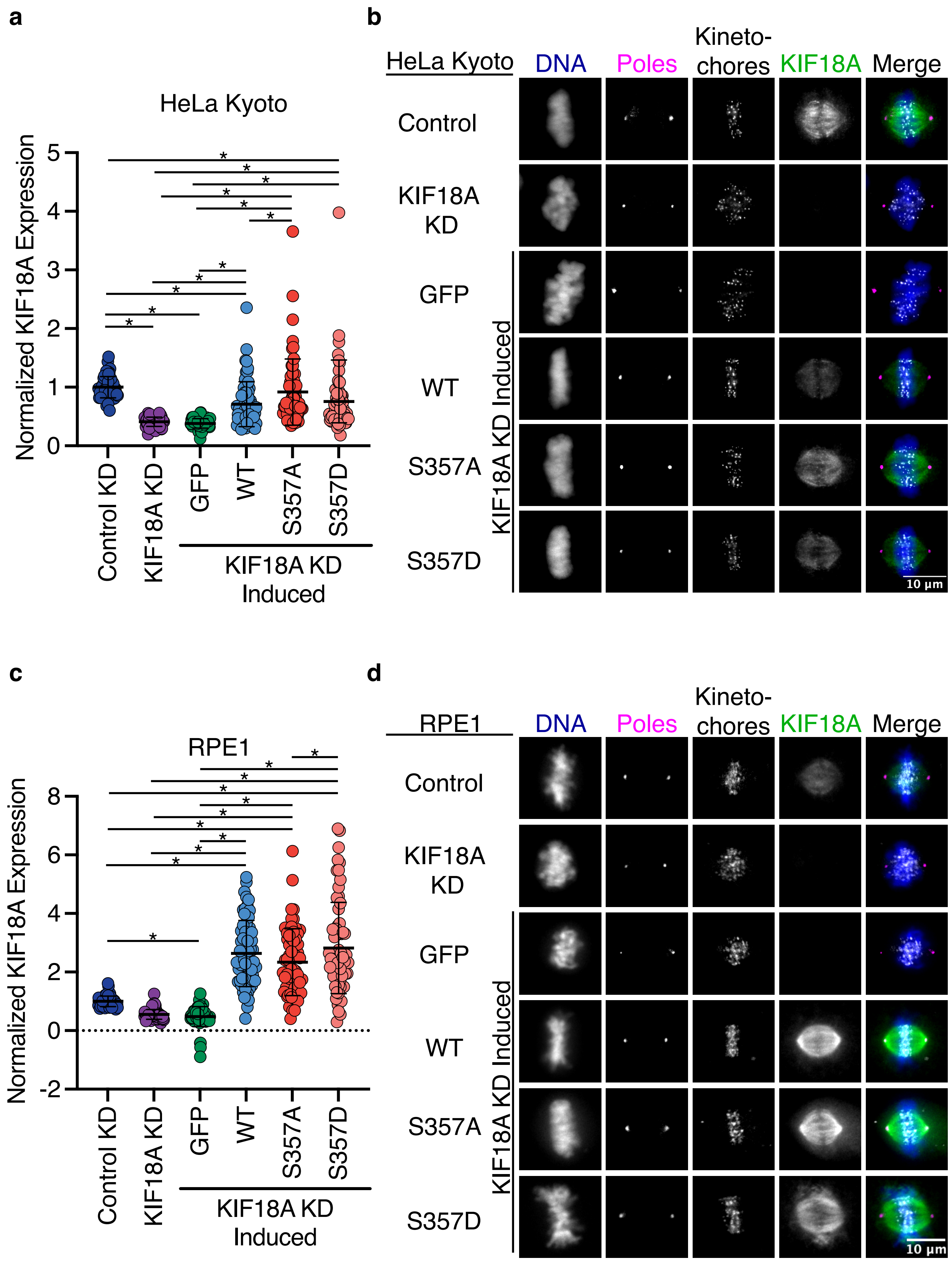

139

bioRxiv preprint doi: https://doi.org/10.1101/2023.05.02.539080; this version posted May 2, 2023. The copyright holder for this preprint (which was not certified by peer review) is the author/funder, who has granted bioRxiv a license to display the preprint in perpetuity. It is made available under aCC-BY 4.0 International license.

Supplementary Figure 1: KIF18A inducible cell lines have similar expression. (a) Quantification of KIF18A expression in HeLa Kyoto cells from KIF18A antibody intensity. Fluorescence values were normalized to the mean control KIF18A intensity. Solid horizontal line indicates mean, vertical lines indicate standard deviation. Each dot represents a single cell. Data were acquired from three experimental replicates. Data were analyzed using a one-way ANOVA with Tukey's test for multiple comparisons. P value style: <0.05 (*), if no significance is indicated result was not significant (> 0.05). **(b)** Representative immunofluorescence images of KIF18A expression in HeLa Kyoto cells. Cells were fixed approximately 24 hours after siRNA treatment to knockdown endogenous KIF18A and induction of GFP-KIF18A with doxycycline. Colors indicate pseudo-color in merged image. Brightness/contrast levels for KIF18A are set to be equivalent across conditions. Brightness/contrast levels for DNA, poles, and kinetochores are set differently to optimize visualization. Scale bar is 10 µm. WT: wild-type. KD: knockdown. **(c)** Quantification of KIF18A expression in RPE1 cells from KIF18A antibody intensity. Fluorescence values were normalized to the mean control KIF18A intensity. Solid horizontal line indicates mean, vertical lines indicate standard deviation. Each dot represents a single cell. Data were acquired from three experimental replicates. Data were analyzed with a one-way ANOVA with Tukey's test for multiple comparisons. P value style: <0.05 (*), if no significance is indicated result was not significant (> 0.05). **(d)** Representative immunofluorescence images of KIF18A expression in RPE1 cells. Cells were fixed approximately 24 hours after siRNA treatment to knockdown endogenous KIF18A and induction of GFP-KIF18A with doxycycline. Colors indicate pseudo-color in merged image. Scale bar is 10 µm. WT: wild-type. KD: knockdown.

bioRxiv preprint doi: https://doi.org/10.1101/2023.05.02.539080; this version posted May 2, 2023. The copyright holder for this preprint (which was not certified by peer review) is the author/funder, who has granted bioRxiv a license to display the preprint in perpetuity. It is made available under aCC-BY 4.0 International license.

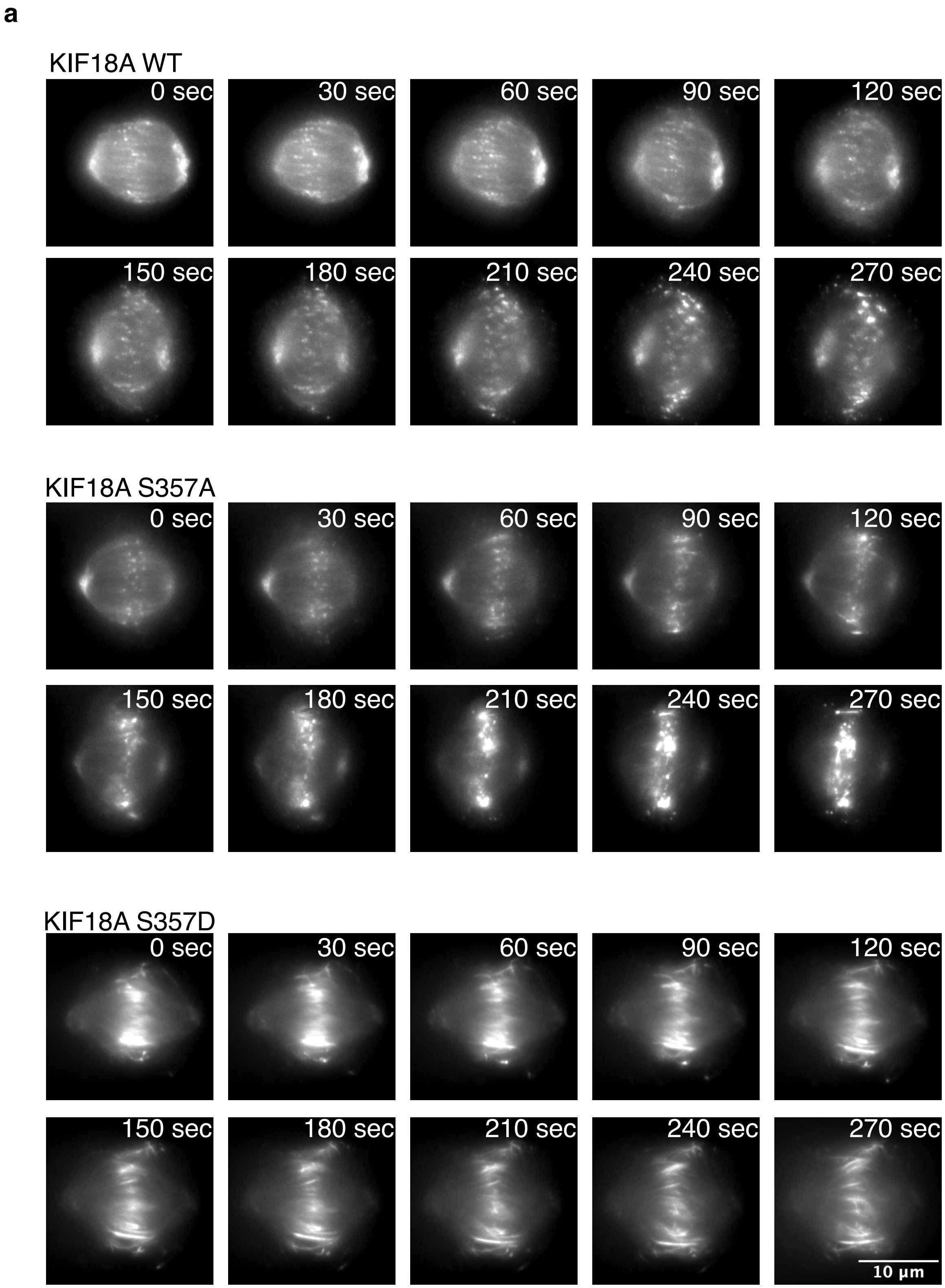

bioRxiv preprint doi: https://doi.org/10.1101/2023.05.02.539080; this version posted May 2, 2023. The copyright holder for this preprint (which was not certified by peer review) is the author/funder, who has granted bioRxiv a license to display the preprint in perpetuity. It is made available under aCC-BY 4.0 International license.

Supplementary Figure 2: Stabilization of microtubules with taxol does not rescue peripheral localization of KIF18A S357D. (a) Representative still images from live-cell imaging of RPE1 GFP-KIF18A inducible cells lines. Cells were imaged approximately 24 hours after siRNA treatment to knockdown endogenous KIF18A and induction of GFP-KIF18A with doxycycline. Immediately prior to imaging, cells were spiked with 10 µM paclitaxel (0 sec) and time stamps indicate elapsed time after taxol treatment. Scale bar is 10 µm. WT: wild-type.

bioRxiv preprint doi: https://doi.org/10.1101/2023.05.02.539080; this version posted May 2, 2023. The copyright holder for this preprint (which was not certified by peer review) is the author/funder, who has granted bioRxiv a license to display the preprint in perpetuity. It is made available under aCC-BY 4.0 International license.

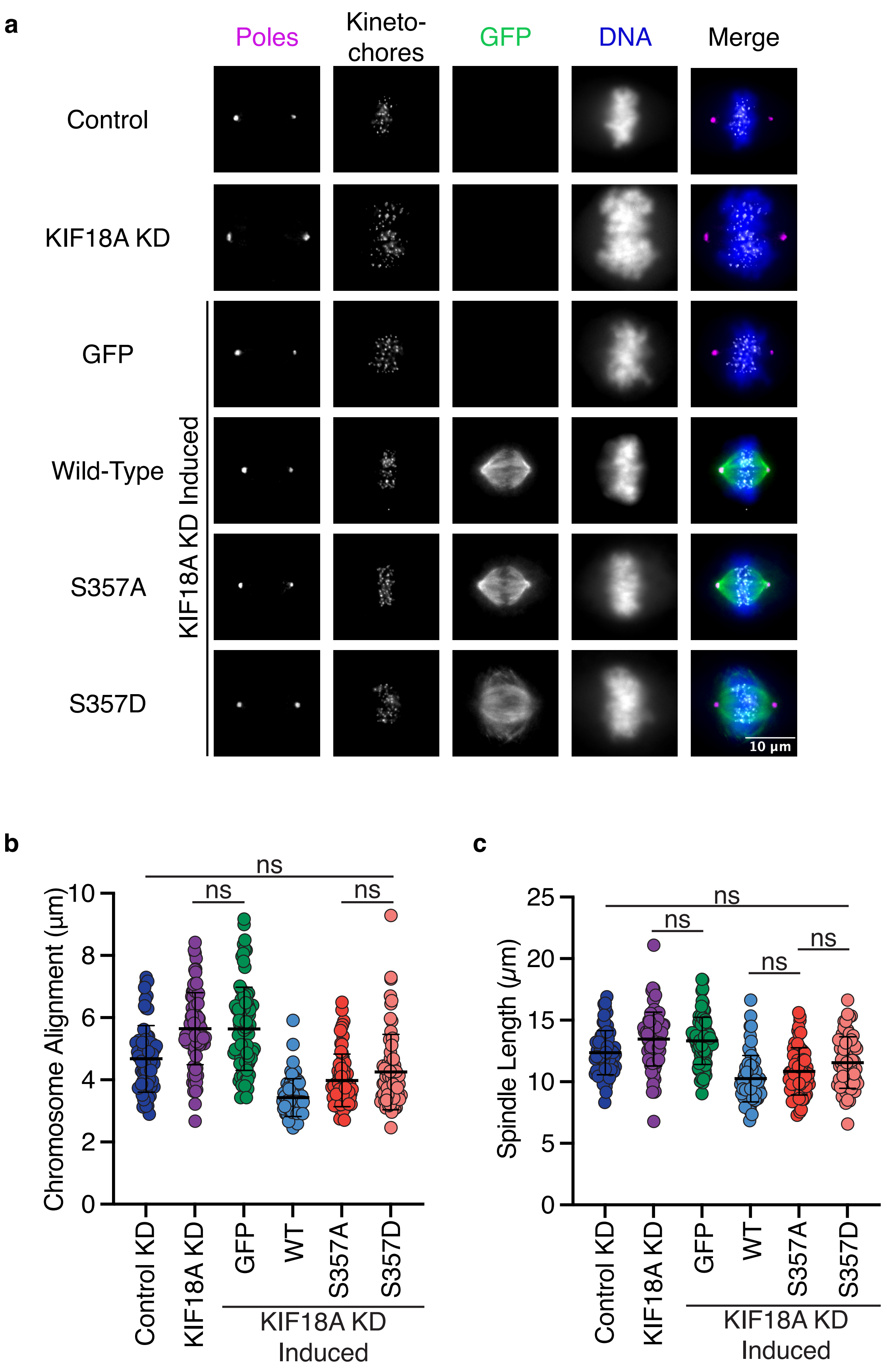

bioRxiv preprint doi: https://doi.org/10.1101/2023.05.02.539080; this version posted May 2, 2023. The copyright holder for this preprint (which was not certified by peer review) is the author/funder, who has granted bioRxiv a license to display the preprint in perpetuity. It is made available under aCC-BY 4.0 International license.

Figure S3: KIF18A S357D displays reduced chromosome alignment and spindle length control in RPE1 cells. (a) Representative immunofluorescence images of RPE1 cells. Cells were fixed approximately 24 hours after siRNA treatment to knockdown endogenous KIF18A and induction of GFP-KIF18A with doxycycline. Color indicates pseudo-color in merged image. Brightness/contrast levels for GFP are set to be equivalent, brightness/contrast levels for poles, kinetochore, and DNA are set differently to optimize visualization. Scale bar is 10 µm. KD: knockdown. **(b)** Quantification of chromosome alignment in RPE1 cells. Chromosome alignment was determined by measuring the distribution of kinetochores between spindle poles in a metaphase cell. This distribution was fit to a gaussian curve and the width of the distribution at half the maximum fluorescence intensity was recorded as the value for chromosome alignment. Solid horizontal line indicates mean, vertical lines indicate standard deviation. Each dot represents a single cell. Data were acquired from three experimental replicates and were analyzed using a one-way ANOVA with Tukey's test for multiple comparisons. P value style: >0.05 (ns), if no significance is indicated result was significant (< 0.05). KD: knockdown, WT: wild-type. **(b)** Quantification of spindle length in RPE1 cells. Solid horizontal line indicates mean, vertical lines indicate standard deviation. Each dot represents a single cell. Data were acquired from three experimental replicates and analyzed using a one-way ANOVA with Tukey's test for multiple comparisons. P value style: >0.05 (ns), if no significance is indicated result was significant (< 0.05). KD: knockdown, WT: wild-type.
